# Supplementary material for: miR-29a-3p Regulates Autophagy by Targeting Akt3-Mediated mTOR in SiO2-Induced Lung Fibrosis
Source: Int J Mol Sci. 2023 Jul 14;24(14):11440. doi: 10.3390/ijms241411440 (PMC10380316; doi:10.3390/ijms241411440)
Supplement: Supplementary file 1 [file ijms-24-11440-s001.zip › ijms-2449231-supplementary.pdf]

Supplementary information.

Supplementary Table S1. The sequences of miR-29a mimic/inhibitor and siR-Akt3

Primer	Sequence
Mmu-miR-29a-3p mimic	sense (5′-3′)-UAGCACCAUCUGAAAUCGGUUA
	antisence (5′-3′)- ACCGAUUUCAGAUGGUGCUAUU
Mmu-miR-29a-3p inhibitor	sense (5′-3′)-UAACCGAUUUCAGAUGGUGCUA
siR-Akt3-144	sense (5′-3′)- GCUCAUUCAUAGGCUAUAATT
	antisence (5′-3′)- UUAUAGCCUAUGAAUGAGCTT
siR-Akt3-818	sense (5′-3′)- GCUUUGGACUAUCUACAUUTT
	antisence (5′-3′)- AAUGUAGAUAGUCCAAAGCTT
siR-Akt3-1315	sense (5′-3′)- GCCUCAAGUAACAUCUGAATT
	antisence (5′-3′)- UUCAGAUGUUACUUGAGGCTT

Supplementary Table S2. The sequences of primer pairs

Primer	Sequence	Amplification
miR-29a-3p	Forward 5′- CTGCCGTAGCACCATCTGA -3′<br>Reverse 5′- TATCCTTGTTCACGACTCCTTCAC-3′	77
U6	Forward 5′- CGCTTCGGCAGCACATATAC-3′<br>Reverse 5′-TTCACGAATTTGCGTGTCATC-3′	87
Akt3	Forward 5′- AGTGGGCATCCAGGTCCTAT -3′<br>Reverse 5′- GGGTGAAAAGCCACCAGACT -3′	127
GAPDH	Forward 5′- CATCACTGCCACCCAGAAGACTG-3′<br>Reverse 5′- ATGCCAGTGAGCTTCCCGTCAG-3′	141

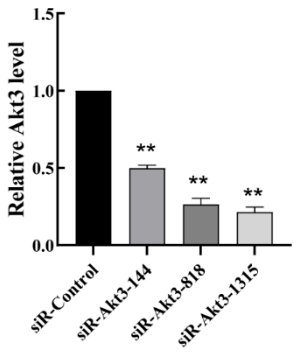

Supplementary Figure S1. qRT-PCR was performed to determine the transfection efficiency of the three siR-Akt3 in TC-1 cells. Data are presented as the mean ± SD from at least three independent experiments; ** indicates p <0.01 vs. the control group.

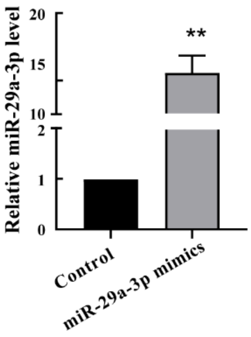

Supplementary Figure S2. qRT-PCR was used to measure the effectiveness of miR-29a-3p mimics and inhibitor transfection in TC-1 cells. Data are presented as the mean ± SD from at least three independent experiments; ** indicates $p < 0.01$ vs. the control group.
